# Matched Analysis of the Risk Assessment and Prediction Tool for Post-Operative Disposition Needs in a Spinal Oncology Population

**DOI:** 10.1177/21925682251414112

**Published:** 2026-02-10

**Authors:** Emily Xu, Ritesh Karsalia, Jason Kost, Kristen Park, Rainer D. Malhotra, Aidan Gor, Scott D. McClintock, Anish Butala, Gabrielle W. Peters, Alvand Hassankhani, Hayley M. Knollman, James Schuster, Colbey W. Freeman, Neil R. Malhotra

**Affiliations:** 1 Department of Neurosurgery, 6572 University of Pennsylvania, Philadelphia, PA, USA; 2 McKenna EpiLog Fellowship in Population Health, 6572 University of Pennsylvania, Philadelphia, PA, USA; 3 Department of Radiology, 6572 University of Pennsylvania, Philadelphia, PA, USA; 4 The West Chester Statistical Institute and Department of Mathematics, West Chester University, West Chester, PA, USA; 5 Department of Radiation Oncology, 6572 University of Pennsylvania, Philadelphia, PA, USA; 6 Division of Hematology and Oncology, 6572 University of Pennsylvania, Philadelphia, PA, USA

**Keywords:** discharge disposition, predictive scale, spinal oncology

## Abstract

**Study Design:**

Retrospective cohort study.

**Objectives:**

As cancer survival improves, metastatic spinal cancer has become increasingly common worldwide. Given the high resource demands of spinal oncology care, tools to optimize perioperative planning are essential. The objective of the study was to assess the effectiveness of the Risk Assessment and Prediction Tool (RAPT) in predicting post-operative needs in patients undergoing surgery for spinal tumors.

**Methods:**

Consecutive patients (n = 384) undergoing spinal oncology surgery were enrolled and prospectively assessed with RAPT. Coarsened exact matching (CEM) was used to retrospectively isolate risk factors associated with outcomes. Enrolled patients with a low RAPT score (≤9, n = 44) were exact matched against high-scoring patients (10-12, n = 44). The primary outcome of interest was post-acute care disposition; secondary outcomes were 30- and 90- day ED visits, readmissions, and reoperations. McNemar’s test was utilized for matched comparisons.

**Results:**

A low RAPT score was significantly associated with non-home discharge (OR = 4.33 [1.23, 15.20], *P* = 0.02) and 30-day readmission (OR = 3.66 [1.02, 13.14], 0.03). Among low-scoring patients, 31.8% required post-acute care (while only 11.3% of high-scoring patients required post-acute care). A low RAPT score was not associated with ER visits, reoperation, or mortality. Isolation of the RAPT walk score alone significantly predicted non-home discharge (OR = 2.8 [1.01, 7.78], *P* = 0.04).

**Conclusions:**

When applied prospectively before spinal cancer surgery, the RAPT tool and its subcomponents effectively predict post-acute care needs. Pre-operative prediction of non-home discharge may help guide in-hospital resource allocation and post-acute care of spinal oncology patients.

## Introduction

Spinal tumors are becoming increasingly common.^1^ Nearly 30% of cancer patients develop spine metastases and require surgical intervention to mitigate symptoms and morbidity.^2^ While surgery can alleviate spinal cord compression, recovery can be associated with overutilization of post-acute resources.^3,4^ A meta-analysis of 13 spine surgery studies found that 30-day readmission rates ranged from 4.2% and 7.4%, with common reasons for readmission including wound infections and medical complications.^4^ Additionally, these patients often utilize high amounts of healthcare resources during the course of their multi-modal treatment.^1,2^ To address these challenges, the Penn SOaR^2^ program (Surgical Spinal Oncology, Medical Oncology, and Radiation Oncology/Radiology) was established to coordinate care for spinal oncology patients. However, predicting which patients can be safely discharged to home, as opposed to a higher level of care, after surgery remains difficult. Therefore, clinical prediction tools are needed to appropriately manage perioperative risk factors and patient discharge planning.

The Risk Assessment and Predictor Tool (RAPT) was initially developed in 2003 to assess the need for post-operative care within orthopedic surgery patients.^3-7^ It is based on six factors: age, gender, walk score, gait assist score, community support score, and home support score. Higher RAPT scores indicate likely home discharge while lower score indicate probable need for further inpatient rehabilitation. Previous studies have illustrated RAPT’s ability to predict post-operative outcomes for patients in several neurosurgical procedures, such as cervical spine surgery and lumbar fusion.^8-10^ However, there is a lack of well-controlled studies into the tool’s effectiveness for spinal oncology patients.

The primary objective of this study is to evaluate the effectiveness of RAPT in predicting post-acute care disposition for patients undergoing spinal tumor surgery. The secondary objective is to determine the effectiveness of RAPT in predicting ER visits, readmissions, and 30- and 90-day reoperation rates. This work has the potential to provide valuable insights into the impact of RAPT on post-operative care for spinal tumor patients and could be used to further enhance the quality of spine surgical care by informing resource allocation in a targeted manner.

## Methods

### Patient Selection

Data was retrospectively collected from 1841 consecutive spine tumor surgeries from 2013-2024 at a multi-hospital academic medical center. Given the retrospective design of the study, patient consent was waived. All included cases were inpatient admissions, utilized general anesthesia, had a documented RAPT score from a pre-operative visit, and surgical pathology confirming neoplasm. Primary and metastatic spine tumors were both included, as well as cases with and without instrumentation. A total of 384 cases were further analyzed (Figure 1).

**Figure 1.**
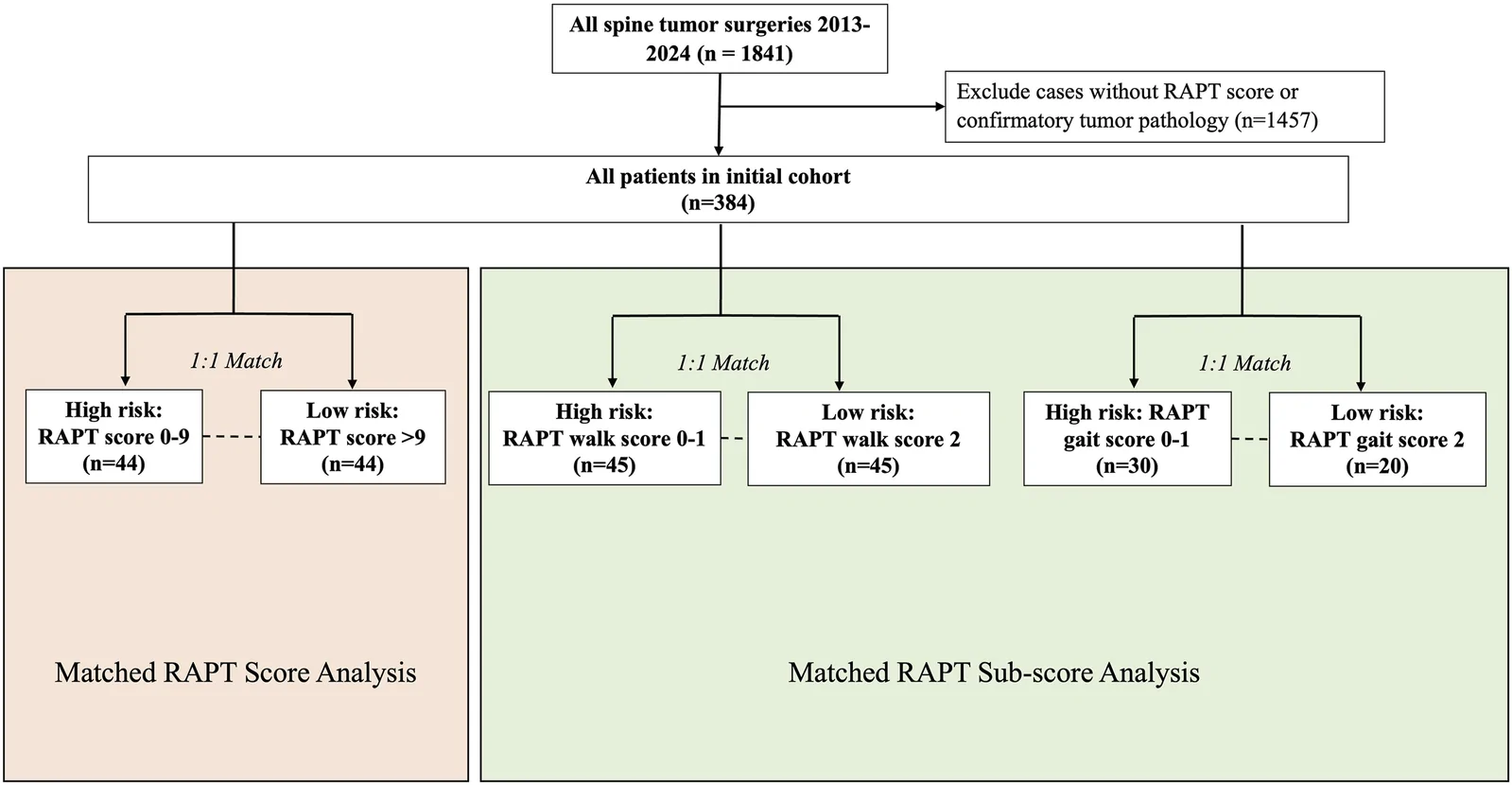
Patient Selection. Flowchart indicating the patients selected for matching for RAPT score and sub-score analysis

### Data Extraction

RAPT scores were prospectively recorded at the pre-operative visit using a standard questionnaire that assesses the patient’s age, gender, baseline ambulation, assistive devices, and presence of support systems. EpiLog,^11^ a non-proprietary software designed for electronic medical record data collection and quality improvement initiatives, was used to collected other patient medical and demographic data, such as sex, American Society of Anesthesiologists (ASA) grade, smoking status, insurance type, surgical history, body mass index (BMI), median household income (MHI), race, and all 16 comorbid conditions assessed in the Charlson Comorbidity Index (CCI). 

The primary post-operative outcome was discharge disposition (home vs non-home). Secondary outcomes included 30- and 90-day post-operative Emergency Department (ED) visits, readmissions, and reoperations following the operation. At post-operative follow-up visits, patients were routinely asked about encounters at outside health systems to allow for documentation and inclusion of these events in the analysis.

This study was approved by the Institutional Review Board at the participating institution (IRB #825431).

### Statistical Analysis and Matching

Patients were clustered into predicted risk cohorts based on RAPT score, as in prior orthopedic research^7^: low-risk (score >9) vs high-risk (score = 0-9). Subsequently, coarsened exact matching (CEM) was performed to generate exact-matched groups among risk cohorts. Low and high-risk patients were matched one-to-one along the following covariates: gender (male vs female),^12^ ASA grade (exact),^13^ smoking history (prior/never),^14^ insurance type (public/private),^15^ prior surgery (binary),^16^ CCI score (<4, 5-6, >7),^17^ BMI (<18.5, 18.5-30, or >30),^18^ race (white/non-white),^19^ and median household income (below/above median).^20^ These criteria were intentionally selected for the matching protocol due to the known effect of these covariates on surgical morbidity and post-operative outcomes.^14,17-19^ We did not match on age due to its inclusion in the RAPT score. Exact matching resulted in a study cohort of 44 matched pairs of patients based on these factors. Sub-analysis of matched cohorts based on the individual RAPT components was similarly done. For the individual RAPT walk scores, patients were grouped by score 0-1 (cannot walk more than 2 blocks) vs 2 (can walk 2+ blocks) and for the RAPT gait scores, patients were grouped by score 0-1 (requires some assistive device to walk) vs 2 (does not require assistance). Matching was conducted with the same covariates as detailed above and 45 exact matches for the walk score analysis were uncovered. Thirty (30) matches for the gait score analysis were uncovered.

Differences in patient demographics and characteristics were assessed using chi-squared and non-parametric tests. Outcomes in the coarsened exact matched analysis were assessed using McNemar’s test and non-parametric testing for categorical and continuous variables, respectively.

## Results

### Demographics

Out of the initial cohort of 384 unmatched patients, 88 patients were in the high-risk group based on RAPT score (score 0-9) and there were 296 patients in the low-risk group (RAPT score >9) (Table 1). Prior to matching, there were significantly more females (68.5% vs 48.7%, *P* < 0.001) and patients with private (63.6% vs 25%, *P* < 0.001) insurance in the high-risk group. These patients also had a higher mean age (63.51 vs 54.17 years, *P* < 0.0001), mean CCI score (6.14 vs 4.38, *P* = 0.0001), and mean ASA score (2.78 vs 2.58, *P* = 0.002). After coarsened exact matching, only average age (63.5 vs 53.4 years, *P* = 0.004) and proportion of patients with history of surgery within the past 30 days (0% vs 9.09%, *P* = 0.04) were significantly different between the high vs low risk groups.

**Table 1.** Patient Characteristics for Pre- and Post-matching Cohorts

	Pre-match			Post-match		
	RAPT score 0-9 (n = 88)	RAPT score >9 (n = 296)	*P*-value	RAPT score 0-9 (n = 44)	RAPT score >9 (n = 44)	*P*-value
Age (mean, range)	63.51 [32-88]	54.17 [16-86]	***P* < 0.0001**	63.5 [(27-84)	53.4 [22-76]	***P* = 0.004**
Gender (n, %)						
Male	27 (31.46%)	152 (51.33%)	***P* < 0.001**	15 (34.09%)	15 (34.09%)	*P* = 1
Female	61 (68.54%)	144 (48.67%)		29 (65.91%)	29 (65.91%)	
Race (n, %)						
White	68 (77.27%)	238 (80.41%)	*P* = 0.5471	37 (84.09%)	37 (84.09%)	*P* = 1
Non-white	20 (22.73%)	58 (19.59%)		7 (15.91%)	7 (15.91%)	
Insurance type (n, %)						
Private	56 (63.64%)	74 (25%)	***P* < 0.001**	21 (47.73%)	21 (47.73%)	*P* = 1
Non-private	32 (36.36%)	222 (75%)		23 (52.27%)	23 (52.27%)	
Prior surgery (n, %)	36 (40.91%)	127 (42.91%)	*P* = 0.739	16 (36.36%)	16 (36.36%)	*P* = 1
Prior surgery in past 30 days (n, %)	3 (3.41%)	19 (6.42%)	*P* = 0.286	0 (0%)	4 (9.09%)	***P* = 0.041**
Tobacco use (n, %)	8 (9.09%)	23 (7.77%)	*P* = 0.689	1 (2.27%)	1 (2.27%)	*P* = 1
Pathology diagnosis						
Breast cancer	4	8	*P* = 0.0982	0	2	*P* = 0.51
Ependymoma	10	25		5	2	
Lung cancer	2	11		1	2	
Meningioma	17	38		9	7	
Schwannoma	14	73		9	14	
Other	41	141		20	17	
ASA score (mean, range)	2.78 [2-4]	2.58 [1-4]	***P* = 0.002**	2.659 [2-3]	2.659 [2-3]	*P* = 1
BMI (mean, range)	28.41 [18.14-48.83]	28.5 [17.51-53.14]	*P* = 0.777	27.9 [19.49-48.83]	28.5 [19.35-53.14]	*P* = 0.76
CCI Score (mean, range)	6.14 [0-17]	4.38 [0-15]	***P* = 0.0001**	4.72 [0-14]	4.40 [0-15]	*P* = 0.38
Median household income, dollars (median, range)	73945 [28046-154808]	75885 [18877-193152]	*P* = 0.2455	74434 [31113-129064]	74708 [18877-122266]	*P* = 0.97
Length of stay, days (mean, range)	6.27 [1.04-59.25]	5.46 [0-68.38]	*P* = 0.0712	5.21 [1.04-29.25]	5.83 [0-48.08]	*P* = 0.56

Statistically significant *p*-values are bolded.

### Outcomes After Matching

After CEM, patients with RAPT score 0-9 were more likely to have a non-home discharge after surgery (OR = 4.33 [1.23, 15.20]), *P* = 0.0213) when compared to matched patients with RAPT score >9 (Table 2, Figure 2), with a sensitivity of 75%, specificity of 57%, and AUC of 0.61. Low RAPT score patients were also more likely to be readmitted within 30 days (OR = 3.66 [1.02, 13.14], *P* = 0.0325) (Table 2), with a sensitivity of 75% and specificity of 56%, and AUC of 0.59. There were no differences in average length of stay (5.21 vs 5.83 days, *P* = 0.56) (Table 2) or 30-day ED visits (OR = 0.6667 [0.188, 2.36], *P* = 0.75) between the two groups. Assessing longer-term outcomes, we also found no differences in 90-day ED visits (OR = 2 [0.602, 6.64], *P* = 0.39), readmissions (OR = 2 [0.6836, 5.85], *P* = 0.30), reoperations (OR = 0.667 [0.114, 3.98], *P* = 1), or mortality (OR = 1 [0.25, 4.00], *P* = 1) (Table 2).

**Table 2.** Surgical Outcomes Based on Matched Patients With RAPT Score 0-9 vs RAPT Score >9

Outcome	OR (95% CI)	*P*-value
Non-home discharge	4.33 [1.234, 15.20]	**0.0213**
30D Readmission	3.66 [1.02, 13.14]	**0.0325**
30D ED visit	0.6667 [0.188, 2.36]	0.7539
30D Reoperation	NA^a^	NA^a^
90D Readmission	2 [0.6836, 5.85]	0.3018
90D ED visit	2 [0.602, 6.64]	0.3877
90D Reoperation	0.667 [0.114, 3.98]	1
90D Mortality	1 [0.25, 4.00]	1

^a^There were not enough observations to calculate an odds ratio.

Statistically significant *p*-values are bolded.

**Figure 2.**
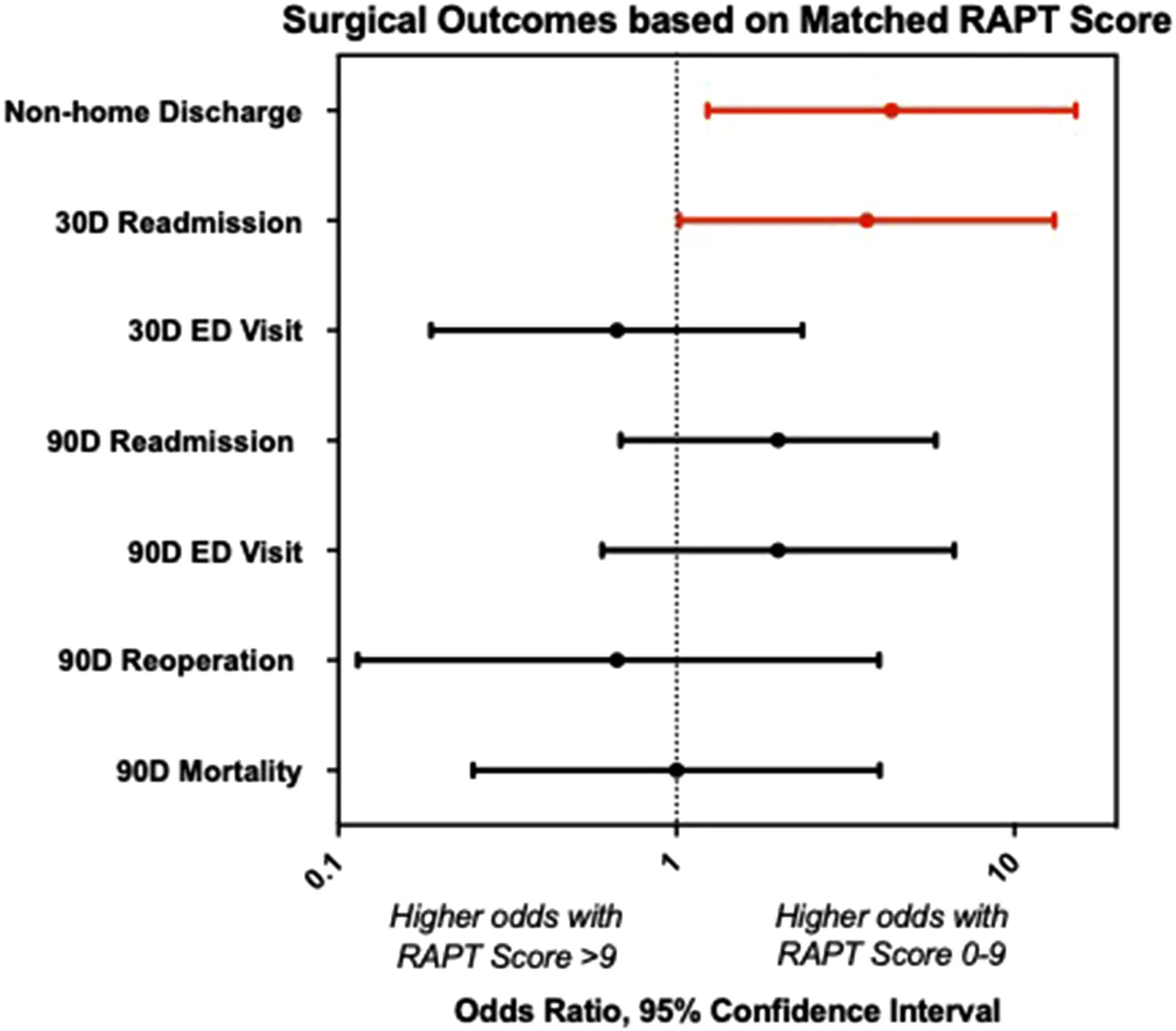
Surgical Outcomes associated with RAPT Score in Matched Patients. Forest plot demonstrating odds ratios of surgical outcomes comparing matched patients with RAPT score 0-9 (high risk group) vs RAPT score >9 (low risk group). Odds ratio >1 indicates that the outcome is more likely in the score 0-9 group, while odds ratio <1 indicates that the outcome is more likely in the score >9 group. Error bars denote 95% confidence intervals and dotted line marks odds ratio of 1. Significant results with *P* < 0.05 are depicted in red

### Matched RAPT Sub-Score Analysis

Analysis of the individual RAPT walk scores among matched patients showed that a walk score of 0-1 increased the chance of a non-home discharge (OR = 2.8 [1.008, 7.775], *P* = 0.0389), compared to otherwise similar patients who had a walk score of 2 (Table 3, Figure 3). There were no differences in 30- or 90- day readmissions (OR = 2 [0.6836, 5.85], *P* = 0.3018; OR = 1.37 [0.55, 3.41], *P* = 0.6476), ED visits (OR = 1 [0.25, 3.99], *P* = 1; OR = 0.83 [0.25, 2.73], *P* = 1), or reoperations (OR = 1 [0.06, 15.9], *P* = 1; OR = 2 [0.366, 10.91], *P* = 0.6875). Mortality after 90 days was also similar between both groups (OR = 1 [0.289, 3.45], *P* = 1). Analysis of RAPT gait scores revealed no differences in non-home discharge (OR = 0.34 [0.115, 1.14], *P* = 0.11), 30-day readmission (OR = 0.25 [0.05, 1.17], *P* = 0.1094), or 30-day ED visits (OR = 0.4 [0.077, 2.06], *P* = 0.4541) when comparing matched patients with gait score 0-1 vs score 2. Furthermore, 90-day outcomes, including readmission (OR = 0.5 [0.1506, 1.66], *P* = 0.3877), ED visits (OR = 0.6 [0.14, 2.51], *P* = 0.72), reoperations (OR = 2 [0.1814, 22.05], *P* = 1) and mortality (OR = 1 [0.25, 4.00], *P* = 1), were also not significantly different based on gait score.

**Figure 3.**
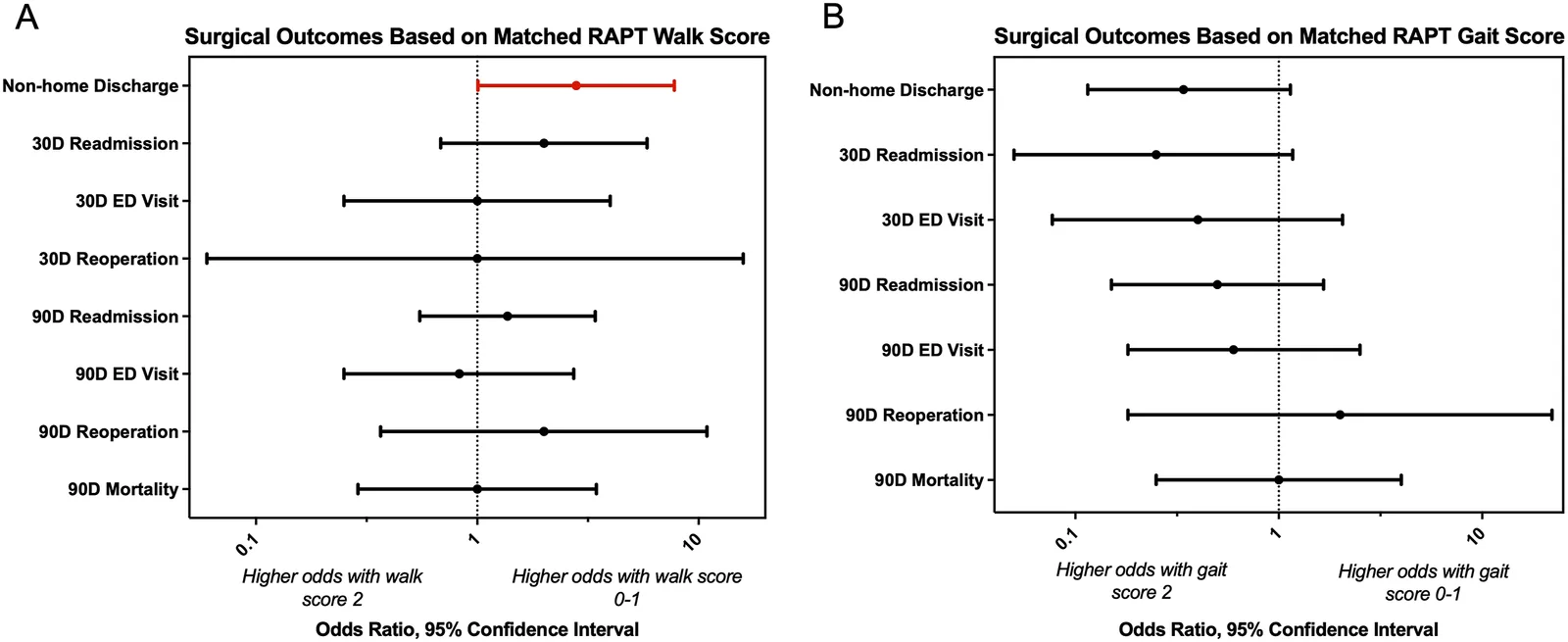
Matched sub-analysis of Individual RAPT Components. Odds of experiencing surgical outcomes in matched patients with (A) RAPT walk score 0-1 (unable to walk more than 2+ blocks) vs 2 (able to walk more than 2+ blocks) and (B) RAPT gait score 0-1 (requires some assistive device to walk) vs score 2 (does not require any device to walk). Odds ratio greater than 1 indicates higher chance of outcome with walk or gait score 0-1. Odds ratios with 95% confidence intervals are shown with significant results highlighted in red

**Table 3.** Surgical Outcomes Based on Matched RAPT Subcomponent Scores

Outcome	RAPT walk score 0-1 vs score 2 (n = 45 matches)		RAPT gait score 0-1 vs Score 2 (n = 30 matches)	
	OR (95% CI)	*P*-value	OR (95% CI)	*P*-value
Non-home discharge	2.8 [1.008, 7.775]	**0.0389**	0.34 [0.115, 1.14]	0.11
30D Readmission	2 [0.6836, 5.85]	0.3018	0.25 [0.05, 1.17]	0.1094
30D ED visit	1 [0.25, 3.99]	1	0.4 [0.077, 2.06]	0.4531
30D Reoperation	1 [0.06, 15.9]	1	NA^a^	NA^a^
90D Readmission	1.37 [0.55, 3.41]	0.6476	0.5 [0.1506, 1.66]	0.3877
90D ED visit	0.83 [0.25, 2.73]	1	0.6 [0.14, 2.51]	0.72
90D Reoperation	2 [0.366, 10.91]	0.6875	2 [0.1814, 22.05]	1
90D Mortality	1 [0.289, 3.45]	1	1 [0.25, 4.00]	1

^a^There were not enough observations to calculate an odds ratio.

Statistically significant *p*-values are bolded.

## Discussion

The RAPT assessment has been a well-validated prediction tool for post-operative discharge disposition in orthopedic surgery.^6-9,12,13^ More recently, studies have demonstrated its application to neurosurgical procedures,^8^ including lumbar fusion,^10^ but whether RAPT can be applied to more complex procedures in medically complicated patients remains unknown. In this study, we assessed the ability of RAPT to predict post-operative outcomes after spinal tumor surgery. We employ a coarsened exact matching protocol to rigorously control for ten variables known to affect neurosurgical outcomes including race, BMI, and CCI score.^14,17-19^ Our study finds that RAPT is able to predict non-home discharge and 30-day readmission after surgery. Furthermore, the RAPT walk score alone was adequate in predicting discharge disposition.

Due to the high resource demands and the morbidity of spinal neoplasms, and their treatment, there is significant interest in developing tools to predict post-operative outcomes. A number of survival prediction tools have been developed, including Baeur, Tokuhashi, and Tomita.^21-23^ The Tokuhashi score takes into account general condition, number and location of metastases, site of the primary cancer and degree of spinal cord palsy.^21^ It has since been revised and has demonstrated favorable accuracy in predicting patient prognosis.^24^ Tomita et al later simplified Tokuhashi’s system and added weighting to each factor.^22^ These two scales are often used in evaluating surgical candidacy. Other scales have focused on short-term post-operative outcomes.^25-27^ Pre-operative patient functional status has been consistently identified as significant predictor in non-home discharge.^28,29^ Ehresman et al found that non-routine discharge in patients undergoing surgery for vertebral column tumors was predicted by emergency admission, increased frailty, serum albumin level, and procedures with multiple stages.^29^ Another study on intradural extramedullary spinal tumors associated older age and increased medical comorbidity burden with greater post-acute care needs.^28^ Our results are largely consistent with these previous studies, as RAPT is essentially evaluating pre-operative functional status in a convenient format. Simplicity of the RAPT permits easy incorporation into the electronic medical record, as exemplified by our research group’s effort to integrate RAPT into our health system’s EMR using an automated program called EpiLog.^8^ Additionally, unlike other scoring tools,^25-27^ RAPT does not require additional lab values, patient imaging, or comprehensive knowledge of the patient’s medical comorbidities. RAPT can be entered by any level of caregiver and even the patients themselves. Automated calculation algorithms support simple tabulation. Therefore, it can be quickly administered at a pre-operative visit with results routed to the pre-procedure note and to the social work team.

Discharge disposition is an important variable of clinical outcome and healthcare costs in the spinal oncology population, as up to 40% of patients experience a non-home discharge.^30,31^ Non-home discharge is associated with higher rates of post-operative mortality, wound complications, and reoperations.^28,32^ Pre-operative identification of high-risk patients offers the possibility to develop personalized post-operative protocols to streamline post-operative care, optimize resource allocation, and improve patient satisfaction. There is evidence that intentionally designed discharge planning pathways can reduce readmission and total post-operative healthcare resource usage after discharge,^33,34^ as discharge delays can account for more than 39% of the hospitalization stay cost.^35^

Clinical implementation of the RAPT questionnaire can help facilitate spinal oncology care by reducing peri-operative morbidity and decreasing cost. Assessing RAPT score at a pre-operative visit can help surgeons to adequately assess surgical risk, while also providing patients with appropriate expectations for their discharge plan. Patients identified at high-risk of non-home discharge can be engaged early by case management and transfer requests can be placed promptly to reduce hospitalization time when patients are simply waiting for a bed at a different facility. Low-risk patients can engage in early mobilization after surgery to facilitate home discharge. Furthermore, our finding that the RAPT walk score alone was able to predict discharge disposition indicates that pre-operative ambulation is an important target for optimization. Walking ability is associated with many dimensions of health, including cardiovascular strength, respiratory capacity, and musculoskeletal tone. For spinal oncology patients, ambulation can also indicate the severity of disease burden and has been associated with survival and perioperative recovery.^36^ Pre-operative referral of patients with low walk scores to physical therapy may increase chances of home discharge when surgical delay is deemed to be safe and further prospective studies are warranted.

### Limitations

This study should be interpreted within the context of its limitations. This study was specifically designed to assess the utility of the RAPT questionnaire and its subcomponents to predict post-operative outcomes. The coarsened exact matching analysis strategy used in this study was employed to isolate and compare patients with similar medical comorbidities and demographic profiles, thereby controlling for factors known to affect post-operative outcomes. This matching protocol comes at the cost of limiting sample size, but the rigorous patient matching criteria preserves the internal validity of the results.

For this analysis, we were unable to match patients by tumor pathology due to limitations in the cohort size. Differences in tumor pathology, including primary and metastatic spinal tumors, are known to dictate long-term patient outcomes and prognosis.^37^ However, patient outcomes in the intermediate post-operative period are largely influenced by pre-operative functional status rather than cancer type.^25,38,39^ Hence, other published discharge disposition prediction models have found no correlation with tumor pathology and omitted this variable.^29^ Pre-operative outcome prediction tools that do include cancer pathology have also failed to demonstrate increased clinical utility over tools that do not include it.^40^ Additionally, from a healthcare quality standpoint, primary and metastatic tumors have been shown to have similar readmission and hospitalization costs.^41^ Therefore, the results of our study are still relevant to pre-operative prediction of outcomes after surgery, including in cases when the exact pathology is unknown. Follow-up study in a large multi-center cohort of spinal oncology patients is warranted to understand the relevance of RAPT in different tumor types.

Finally, RAPT was designed to predict discharge disposition, so it has limited predictive ability for other postoperative outcomes. Incorporation of other patient-specific measures, such as frailty, may increase its utility for the spinal oncology population outside the immediate postoperative period.

## Conclusion

Reliable and accurate pre-operative determination of discharge disposition can help facilitate the care of spinal oncology patients, as well as reduce cost. The total RAPT score and the individual walk score can predict discharge disposition following surgery, even after matching patients on multiple covariates known to affect clinical outcomes. Our results provide the rationale for further prospective, randomized studies on the implementation of RAPT in the spinal oncology population.

## References

[1] Sacino AN Chen H Sahgal A , et al. Stereotactic body radiation therapy for spinal metastases: a new standard of care. Neuro Oncol. 2024;26(12 Suppl 2):S76-s87. doi:10.1093/neuonc/noad225 PMC10911798 38437670

[2] Furlan JC Wilson JR Massicotte EM Sahgal A Fehlings MG . Recent advances and new discoveries in the pipeline of the treatment of primary spinal tumors and spinal metastases: a scoping review of registered clinical studies from 2000 to 2020. Neuro Oncol. 2022;24(1):1-13. doi:10.1093/neuonc/noab214 PMC8730766 34508647

[3] Dauty M Schmitt X Menu P Rousseau B Dubois C . Using the risk assessment and predictor tool (RAPT) for patients after total knee replacement surgery. Ann Phys Rehabil Med. 2012;55(1):4-15. doi:10.1016/j.rehab.2011.10.006 22177789

[4] Tan C Loo G Pua YH , et al. Predicting discharge outcomes after total knee replacement using the risk assessment and predictor tool. Physiotherapy. 2014;100(2):176-181. doi:10.1016/j.physio.2013.02.003 23830717

[5] Hansen VJ Gromov K Lebrun LM Rubash HE Malchau H Freiberg AA . Does the risk assessment and prediction tool predict discharge disposition after joint replacement? Clin Orthop Relat Res. 2015;473(2):597-601. doi:10.1007/s11999-014-3851-z PMC4294888 25106801

[6] Konopka JF Hansen VJ Rubash HE Freiberg AA . Risk assessment tools used to predict outcomes of total hip and total knee arthroplasty. Orthop Clin N Am. 2015;46(3):351-362. doi:10.1016/j.ocl.2015.02.004 26043049

[7] Oldmeadow LB McBurney H Robertson VJ . Predicting risk of extended inpatient rehabilitation after hip or knee arthroplasty. J Arthroplast. 2003;18(6):775. doi:10.1016/s0883-5403(03)00151-7 14513453

[8] Piazza M Sharma N Osiemo B , et al. Initial assessment of the risk assessment and prediction tool in a heterogeneous neurosurgical patient population. Neurosurgery. 2019;85(1):50-57. doi:10.1093/neuros/nyy197 29788192

[9] Berger I Piazza M Sharma N , et al. Evaluation of the risk assessment and prediction tool for postoperative disposition needs after cervical spine surgery. Neurosurgery. 2019;85(5):E902-e909. doi:10.1093/neuros/nyz161 31134280

[10] Glauser G Piazza M Berger I , et al. The risk assessment and prediction tool (RAPT) for discharge planning in a posterior lumbar fusion population. Neurosurgery. 2020;86(2):E140-e146. doi:10.1093/neuros/nyz419 31599332

[11] Gawande A . Why doctors hate their computers. New Yorker.

[12] Karsalia R Gallagher R Borja A , et al. Disparities attributable to sex differences in 4680 lumbar fusion outcomes. World Neurosurg. 2024;12/20:123586. doi:10.1016/j.wneu.2024.123586 39710198

[13] Schoenfeld AJ Ochoa LM Bader JO Belmont PJ . Risk factors for immediate postoperative complications and mortality following spine surgery: a study of 3475 patients from the national surgical quality improvement program. J Bone Joint Surg Am. 2011;93(17):1577-1582. doi:10.2106/jbjs.J.01048 21915571

[14] Turan A Mascha EJ Roberman D , et al. Smoking and perioperative outcomes. Anesthesiology. 2011;114(4):837-846. doi:10.1097/ALN.0b013e318210f560 21372682

[15] Simon RC Kim J Schmidt S , et al. Association of insurance type with inpatient surgery 30-Day complications and costs. J Surg Res. 2023;282:22-33. doi:10.1016/j.jss.2022.09.006 PMC11542174 36244224

[16] Mannion AF Elfering A . Predictors of surgical outcome and their assessment. Eur Spine J. 2006;15(Suppl 1):S93-108. doi:10.1007/s00586-005-1045-9 PMC3454547 16320033

[17] Shinonara K Ugawa R Arataki S Nakahara S Takeuchi K . Charlson comorbidity index is predictive of postoperative clinical outcome after single-level posterior lumbar interbody fusion surgery. J Orthop Surg Res. 2021;16(1):235. doi:10.1186/s13018-021-02377-7 PMC8008557 33785033

[18] Alsoof D Johnson K McDonald CL Daniels AH Cohen EM . Body mass index and risk of complications after posterior lumbar spine fusion: a matched cohort analysis investigating underweight and Obese patients. J Am Acad Orthop Surg. 2023;31(7):e394-e402. doi:10.5435/jaaos-d-22-00667 36525561

[19] Seicean A Seicean S Neuhauser D Benzel EC Weil RJ . The influence of race on short-term outcomes after laminectomy and/or fusion spine surgery. Spine. 2017;42(1):34-41. doi:10.1097/brs.0000000000001657 27128387

[20] Barrie U Montgomery EY Ogwumike E , et al. Household income as a predictor for surgical outcomes and opioid use after spine surgery in the United States. Glob Spine J. 2023;13(8):2124-2134. doi:10.1177/21925682211070823 PMC10538313 35007170

[21] Tokuhashi Y Matsuzaki H Toriyama S Kawano H Ohsaka S . Scoring system for the preoperative evaluation of metastatic spine tumor prognosis. Spine. 1990;15(11):1110-1113. 10.1097/00007632-199011010-00005 1702559

[22] Tomita K Kawahara N Kobayashi T Yoshida A Murakami H Akamaru T . Surgical strategy for spinal metastases. Spine. 2001;26(3):298-306. 10.1097/00007632-200102010-00016 11224867

[23] Bauer H Tomita K Kawahara N Abdel-Wanis ME Murakami H . Surgical strategy for spinal metastases. Spine. 2002;27(10):1124-1126. 10.1097/00007632-200205150-00027 12004183

[24] Tokuhashi Y Ajiro Y Umezawa N . Outcome of treatment for spinal metastases using scoring system for preoperative evaluation of prognosis. Spine. 2009;34(1):69-73. 10.1097/BRS.0b013e3181913f19 19127163

[25] Schoenfeld AJ Le HV Marjoua Y , et al. Assessing the utility of a clinical prediction score regarding 30-day morbidity and mortality following metastatic spinal surgery: the New England spinal metastasis score (NESMS). Spine J. 2016;16(4):482-490. doi:10.1016/j.spinee.2015.09.043 26409416

[26] De la Garza Ramos R Goodwin CR Jain A , et al. Development of a metastatic spinal tumor frailty index (MSTFI) using a nationwide database and its association with inpatient morbidity, mortality, and length of stay after spine surgery. World Neurosurg. 2016;95:548-555. doi:10.1016/j.wneu.2016.08.029 27544340

[27] Elsamadicy AA Koo AB Reeves BC , et al. Hospital frailty risk score and healthcare resource utilization after surgery for metastatic spinal column tumors. J Neurosurg Spine. 2022;37(2):241-251. doi:10.3171/2022.1.SPINE21987 35148505

[28] Ahn A Phan K Cheung ZB White SJW Kim JS Cho SK-W . Predictors of discharge disposition following laminectomy for intradural extramedullary spinal tumors. World Neurosurg. 2019;123:e427-e432. doi:10.1016/j.wneu.2018.11.183 30500579

[29] Ehresman J Pennington Z Feghali J , et al. Predicting nonroutine discharge in patients undergoing surgery for vertebral column tumors. J Neurosurg Spine. 2021;34(3):364-373. doi:10.3171/2020.6.SPINE201024 33254138

[30] Sharma M Sonig A Ambekar S Nanda A . Discharge dispositions, complications, and costs of hospitalization in spinal cord tumor surgery: analysis of data from the United States nationwide inpatient sample, 2003-2010. J Neurosurg Spine. 2014;20(2):125-141. doi:10.3171/2013.9.Spine13274 24286530

[31] Yoshihara H Yoneoka D . Trends in the surgical treatment for spinal metastasis and the in-hospital patient outcomes in the United States from 2000 to 2009. Spine J. 2014;14(9):1844-1849. doi:10.1016/j.spinee.2013.11.029 24291034

[32] Legner VJ Massarweh NN Symons RG McCormick WC Flum DR . The significance of discharge to skilled care after abdominopelvic surgery in older adults. Ann Surg. 2009;249(2):250-255. 10.1097/SLA.0b013e318195e12f 19212178

[33] Kennedy L Neidlinger S Scroggins K . Effective comprehensive discharge planning for hospitalized Elderly1. Gerontol. 1987;27(5):577-580. doi:10.1093/geront/27.5.577 3678895

[34] Naylor M Brooten D Jones R Lavizzo-Mourey R Mezey M Pauly M . Comprehensive discharge planning for the hospitalized elderly. A randomized clinical trial. Ann Intern Med. 1994;120(12):999-1006. doi:10.7326/0003-4819-120-12-199406150-00005 8185149

[35] Du BM Donceel P . Outcome and cost of spinal fractures and spinal tumors. Eur Spine J. 2010;19(Suppl 1):S74. doi:10.1007/s00586-009-1115-5 PMC2899716 19669805

[36] Shamji MF Cook C Tackett S Brown C Isaacs RE . Impact of preoperative neurological status on perioperative morbidity associated with anterior and posterior cervical fusion. J Neurosurg Spine. 2008;9(1):10. doi:10.3171/spi/2008/9/7/010 18590405

[37] Walha S Fairbanks SL . Spinal cord tumor surgery. Anesthesiol Clin. 2021;39(1):139-149. doi:10.1016/j.anclin.2020.11.012 33563377

[38] De la Garza Ramos R Goodwin CR Jain A , et al. Development of a metastatic spinal tumor frailty index (MSTFI) using a nationwide database and its association with inpatient morbidity, mortality, and length of stay after spine surgery. World Neurosurg. 2016;95:548-555. doi:10.1016/j.wneu.2016.08.029 27544340

[39] Verlaan JJ Choi D Versteeg A , et al. Characteristics of patients who survived < 3 months or > 2 years after surgery for spinal metastases: can we avoid inappropriate patient selection? J Clin Oncol. 2016;34(25):3054-3061. doi:10.1200/jco.2015.65.1497 PMC6366641 27400936

[40] Cassidy JT Baker JF Lenehan B . The role of prognostic scoring systems in assessing surgical candidacy for patients with vertebral metastasis: a narrative review. Glob Spine J. 2018;8(6):638-651. doi:10.1177/2192568217750125 PMC6125937 30202719

[41] Lau D Chan AK Theologis AA , et al. Costs and readmission rates for the resection of primary and metastatic spinal tumors: a comparative analysis of 181 patients. J Neurosurg Spine. 2016;25(3):366-378. doi:10.3171/2016.2.SPINE15954 27129043

